# Patient-reported outcome after fast-track hip arthroplasty: a prospective cohort study

**DOI:** 10.1186/1477-7525-8-144

**Published:** 2010-11-30

**Authors:** Kristian Larsen, Torben B Hansen, Kjeld Søballe, Henrik Kehlet

**Affiliations:** 1 The Orthopaedic Research Unit, Department of Orthopedics, Holstebro Regional Hospital, Hospital Unit West, Denmark; 2 The Lundbeck Center for Fast-track Hip and Knee Surgery; 3 Department of Orthopedics, University of Aarhus, Aarhus, Denmark; 4 Section of Surgical Pathophysiology, Rigshospitalet, Copenhagen University, Denmark

## Abstract

**Background:**

A fast-track intervention with a short preoperative optimization period and short postoperative hospitalization has a potential for reduced convalescence and thereby a reduced need for postoperative rehabilitation. The purpose of this study was to describe patient-related outcomes, the need for additional rehabilitation after a fast-track total hip arthroplasty (THA), and the association between generic and disease specific outcomes.

**Methods:**

The study consisted of 196 consecutive patients of which none received additional rehabilitation beyond an instructional exercise plan at discharge, which was adjusted at one in-patient visit. The patients filled in 3 questionnaires to measure health-related quality-of-life (HRQOL) and hip specific function (EQ-5 D, SF36, and Harris Hip Score (HHS)) at 2 time points pre- and 2 time points postoperatively. The observed results were compared to normative population data for EQ-5 D, SF36, and HHS.

**Results:**

3-months postoperatively patients had reached a HRQOL level of 0.84 (SD, 0.14), which was similar to the population norm (*P *= 0.33), whereas they exceeded the population norm at 12 months postoperatively (*P *< 0.01). For SF36, physical function (PF) was 67.8 (SD, 19.1) 3 months postoperatively, which was lower than the population norm (*P *< 0.01). PF was similar to population norm 12-months postoperatively (*P *= 0.35). For HHS, patients never reached the population norm within 12 months postoperatively. Generic and disease specific outcomes were strongly associated.

**Conclusions:**

If HRQOL is considered the primary outcome after THA, the need for additional postoperative rehabilitation for all THA patients following a fast-track intervention is questionable. However, a pre- or early postoperative physical intervention seems relevant if the PF of the population norm should be reached at 3 months. If disease specific outcome is considered the primary outcome after fast-track THA, clear goals for the rehabilitation must be established before patient selection, intervention type and timing of intervention can be made.

## Background

The purpose of a patient receiving THA is to reduce pain and regain health, and WHO proposes to focus on health-related quality-of-life (HRQOL) in the "bone and joint decade" (2000-2010), when monitoring the effect of health care interventions [1]. Therefore the ultimate goal for THA must be to regain HRQOL comparable to the age and gender specific population. Normative data for HRQOL by using generic instruments exist for the questionnaires EuroQOL (EQ-5D) [2,3] and The Medical Outcome Study 36-item Short-Form Health Survey (SF36) [4] which are proven to be useful and validated tools [5-8]. Likewise, reference values from disease specific instruments such as Western Ontario and McMaster Universities Osteoarthritis Index (WOMAC) score and Harris Hip Score (HHS) are available [9], useful and validated tools [6,10-12]. Using EQ-5 D as the reference outcome for HRQOL, the age and gender matched population will show a very small average decrease of 0.01 point in HRQOL from the age of 65 to 70 [2]. In contrast, the THA patient will show a steady and large decrease in HRQOL from onset of hip pain until referral, where the average HRQOL is 0.47 [13]. In a conventional patient path the patient will encounter waiting time from referral until operation, during which their HRQOL will continue the decline [14], and the patient may not reach the population level for HRQOL within the first year [15].

In Denmark approximately 7.000 primary elective total hip arthroplasties (THA) were performed in 30 public hospitals in 2007 [16]. Of these hospitals only 2 (7%) used a "fast-track" intervention defined as preoperative optimization of ≤ 8 weeks or waiting time ≤ 4 weeks, a perioperative intervention reaching discharge criteria ≤ 4 days, and a postoperative intervention focused on information of restrictions and instructions in home exercises in order to achieve normal daily functions as soon as possible in order to reach a health-related quality-of-life (HRQOL) at the population level ≤ 3 months postoperatively [16]. In a fast-track context the population level of HRQOL should be achieved as fast as possible and with as less pain and risk of complications as possible [17,18]. In the study by Larsen et al. [15] THA patients who followed an fast-track intervention reached the age and gender specific HRQOL level of 0.87 at 12 weeks postoperatively. The perioperative intervention followed the general fast-track regimen proposed by Kehlet et al. [17,18], and the THA specific regimen proposed by Husted et al., and Larsen et al. [13,18-22]. The postoperative intervention focused on information of restrictions and instructions in home exercises in order to achieve normal daily functions as soon as possible.

The purpose of this study was to describe patient-related functional outcomes after fast-track THA, the need for additional rehabilitation, and to describe the associations between generic and disease specific outcomes.

## Methods

The study group consisted of consecutive patients fulfilling inclusion criteria for case mix group, who were operated on at the Regional Hospital Holstebro in 2007 and then followed for 12 months postoperatively. The case mix group inclusion criteria were age at or above 55 years and a diagnosis of primary arthrosis. Patients with bilateral disease who were operated on the bilateral hip during the following 12 months were excluded.

The procedures followed in this study were in accordance with the Helsinki Declaration of 1975, as revised in 2000. The study was generally approved by the local research ethics committee, and no further specific approval was demanded because the study is an outcome study, which according to the Danish law "Act on a Biomedical Research Ethics Committee System and the Processing of Biomedical Research Projects", Part 3 **"***Notification and authorization"*: Questionnaire-based projects and register research projects shall only be notified to a regional committee if the project also involves human biological material. The study was registered in The Danish data Protection Agency (j.nr. 2007-41-1197).

### Fast-track intervention

#### Preoperatively

All included patients followed a preoperative optimization regimen, where patients were screened by a nurse on the day of diagnosis using a preoperative arthroplasty screening questionnaire (PASQ) consisting of five areas: 1) nutrition, 2) general health and medication, 3) physical activity, 4) smoking habits, and 5) alcohol consumption. The data for PASQ are derived from 2 sources, a mailed questionnaire and a structured interview included in a motivational conversation. The nurse proposed an intervention plan for all patients with identified risk factors. All patients, accompanied by one relative, were invited to an information and preparation day one week before surgery. The purpose of the information day was to introduce the patients to team staff members, to inform the patients about the fast-track protocol, and to give individual consultation with surgeon, anesthetist, and nurse. The patients were informed about the goals during the hospital stay with intended reach of discharge criteria within 4 nights postoperatively. In addition they were taught pain relief modalities, mobilization strategies, and instruction in use of walking aids.

#### Perioperatively

##### Surgery

All surgery took place in the beginning of the week. Five experienced surgeons performed all operations. Templating was used for implant size. Patients had surgical and anesthetic procedures that followed Danish guidelines of which one is use of cemented implants in THA patients above 70 years of age [23]. We used a medium size posterior incision and a peroperative local infiltration analgesia (LIA) consisting of 100 ml of ropivacaine (Naropin^® ^2 mg/ml), 1 ml ketorolac (Toradol^®^) (30 mg/ml), 0.5 ml adrenaline (1 mg/ml) [24]. Drains were not used. Blood transfusion was standardized, and for antithrombotic prophylaxis we used Arixtra^® ^(Fondaparinux). To prevent infections we used Diclosil^® ^(dicloxacilline) 1 g preoperatively and 3 times postoperatively during the first 24 hours after surgery.

##### Care in specialized ward

The patients were hospitalized in the nurse-led fast-track care unit, which was placed in a separate part of the ward. One nurse was in charge of a team of healthcare professionals who were trained to initiate mobilization activities aggressively. Patients were asked to wear their own clothes during the entire hospital stay to avoid a sense of sickness or dependency, and mobilized in teams. The staff and patients followed daily preset written goals regarding: 1) general information, 2) pain relief, 3) nausea control, 4) nutrition, 5) mobilization, and 6) bowel regulation. Mobilization started on the day of surgery. On the first postoperative day, the goal was to be out of bed 4 hours, including training with physiotherapist and occupational therapist and 8 hours of mobilization/day for the rest of the hospital stay. Detailed description of the accelerated protocol has been published before [13,15,21,22].

##### Mobilization

Physiotherapy and occupational therapy was given once daily on weekdays. Mobilization consisted of all activities out of bed (70% of mobilization time), gait training (15% of mobilization time), and exercises (15% of mobilization time). The physiotherapist was responsible for coaching the patient during exercises and gait training. Exercises focused on strengthening hip and knee muscles and how to avoid restricted movements. When performing exercises there was much focus on intensity, number of repetitions and progression. The patients were taught how to increase exercise and gait training after discharge. The occupational therapist was responsible for instruction regarding performance of personal needs for the THA patients. All patients were given an instructional exercise plan at discharge, which was presented and used at the preoperative information day and during hospitalization.

##### Pain relief

Preoperatively, paracetamol 1 g was given 2-3 hours before the operation. Intraoperatively, we infiltrated 100 mL ropivacaine 2 mg/mL (Naropine) with 1 mL ketorolac 30 mg/mL (Toradol) and 0.5 mL epinephrine 1 mg/mL (adrenaline) Postoperatively, a bolus in the wound catheter was given 8 hours postoperatively consisting of 20 ml of Naropin^® ^(7.5 mg/ml), 1 ml Toradol^® ^(30 mg/ml), 0.5 ml adrenaline (1 mg/ml) [24]. On the day of operation and the first day postoperatively we used paracetamol 1g 4 times per day, and Oxycontin^® ^(oxycodon) (10 mg 2 times daily for patients < 70 years, and 20 mg 2 times daily for ≥ 70 years of age) and if VAS > 3 at rest and/or >5 at mobilization Oxynorm^® ^(oxycodon) 5 mg was given on request. From the second postoperative day, we used paracetamol 1g 4 times per day, Mandolgin^® ^(Tramodol) 50-100 mg 2 times per day and Oxynorm^® ^(Oxycodon) 5 mg if VAS > 3 at rest and/or >5 at mobilization.

##### Discharge criteria

All patients were discharged to home. The discharge criteria were: Absence of any signs of wound problems; satisfactory pain control on oral analgesics; aware of procedures for safely ending medication; knowledge of restrictions; being able to walk safely with or without walking aids; ability to walk up and down stairs; ability to perform home exercises; knowing how to increase home exercises; being able to perform personal care; acceptance of discharge.

#### Postoperatively

##### Restrictions

To avoid dislocation of the hip prosthesis patients were told to avoid flexion of the hip joint beyond 90°, and adduction and internal rotation during the first 3 months. Patients were also taught which positions and activities which could be potentially harmful for the prosthesis.

##### Intervention

In the postoperative intervention period, the patients were invited to an in-patient visit 7 weeks postoperatively, where their status was analyzed and their instructional exercise plan adjusted. No further rehabilitation was made.

### Outcome measures

As part of daily monitoring of outcome for all THA patients operated on at the Hospital Unit West, Denmark, all patients filled in 3 questionnaires (EQ-5 D, SF36, and HHS) at 4 time points (preoperatively at day of diagnosis, preoperatively at the information day, postoperatively at 3-months and at 12-months follow-up). EQ-5 D and SF36 is available in translated and validated Danish versions [2,4]. The HHS questionnaire we used was the self-report HHS (SRHHS) developed by Mahomed et al. [25]. SRHHS is a 7-item questionnaire using the pain and disability items from the original 15-item HHS. The Danish version of SRHHS was translated from English to Danish in respect to the question introduction for the 7 items, and we used the same order of questions, as was reported in the original study by Mahomed et al. [25]. We, however, used the Danish answer categories, which is used in the Danish version of HHS by the Danish Hip Arthroplasty Register http://www.dhr.dk/HofteskemaA2008-pdf/Holstebro.pdf.

### Statistics

The observed results for the 3 questionnaires were compared with normative population data for EQ-5 D, SF-36 and HHS. Normative data for HRQOL by using EQ-5 D were calculated from Sørensen et al. from our observed gender and age data combined with their reported HRQOL data for gender and age groups [3]. Normative data for HRQOL in 8 dimensions with SF 36 were likewise estimated from our observed gender and age data combined with their reported HRQOL data for gender and age groups in Danish Manual for SF36 [4]. A clinically relevant difference in HRQOL score was set at 3 percent point [26]. Primary relevant dimension to encounter the need for additional postoperative rehabilitation was the dimension of physical function (PF) in SF36. The norm data for HHS were obtained using a modified version of HHS (MHHS) by Lieberman et al. where the patients were given no impairment, and the total scores in MHHS were rescaled to 100 points as best score [9]. The maximum score of 90 in SRHHS was rescaled to 100 points for the best score in order to compare the results from two HHS outcome measures [25].

EQ-5 D, PF score in SF36, and HHS at baseline were grouped into high and low score by dividing them at the median score in order to investigate if preoperative score influenced on postoperative HRQOL and physical function at follow-up. Differences between observed score and population score were tested with one-sample t test or two-sample t test. Significance level was set at *P *< 0.05.

To test the association between HHS and HRQOL (EQ-5 D and PF) at 3 and 12-months follow-up we used Spearman's correlation and linear regression of the continuous variables together with multivariate regression by stepwise model building [27]. The 7 items in HHS were dichotomized in a clinically meaningful way. Item 1 was dichotomized at no or mild pain against worse. Item 2 was dichotomized at no cane, or cane for long walks against other answer categories. Item 3 was dichotomized at no or slight limping against moderate or severe limping. Item 4 was dichotomized at walking &8805; 1.5 km against less. Item 5 was dichotomized at climbing stairs normally or by need of banister or cane against other answer categories. Item 6 was dichotomized at can easily put on socks and shoes against can with difficulty or cannot. Finally, item 7 was dichotomized at to sit comfortably in any chair against other answer categories. Step one in the multivariate analysis was a univariate analysis of all variables. Any variable that had a *P*-value of < 0.25 was a candidate for the multivariable model. Step two was a multivariate analysis including all selected candidates. Step three was exclusion of non-contributing variables, and fitting of new models without these non-contributing variables. The variables were excluded one at a time with the variable with the highest *P*-value first, until only variables with a *P*-values of *P *< 0.05 remained in the model. After inspection of the residuals in the preliminary final model of the multivariate linear regression and if sign of no misfit it was then considered to be the final model, which was estimated by using R^2^.

## Results

### Patient sample

A total of 234 patients were eligible for the study, 38 (16%) patients did not meet the inclusion criteria, leaving 196 (84%) patients in the inter-hospital case mix to be included in the study of which 107 (55%) were men with a mean age of 70 yrs (SD 8.3), and 112 (57%) received an un-cemented implant.

The average preoperative optimization period was 46 days (SD, 33). The average hospitalization period was 3.3 days (SD, 2.0), not including 1 patient who was hospitalized 39 days due to complications. A total of 167 of 196 (85%) completed the 3-months follow-up questionnaire. A total of 151 of 196 (77%) completed the 12-months questionnaire. Only 9 of 196 (5%) patients did not complete any of the two follow-up questionnaires. No clinically relevant or significant differences were observed between patients who responded to the two follow-up periods, and patients who were lost to follow-up for age (*P *≥ 0.31), gender (*P *&8805; 0.22), implant type (*P *≥ 0.15), or optimization period (*P *≥ 0.12).

### Health-related quality-of-life

The age and gender matched population mean HRQOL estimated by using EQ-5 D was 0.85. The mean HRQOL for the patient sample preoperatively at time of diagnosis was 0.56 (SD, 0.23), significantly lower than the mean HRQOL preoperatively at the information day 0.59 (SD, 0.23) (*P *< 0.01).

**Figure 1:**
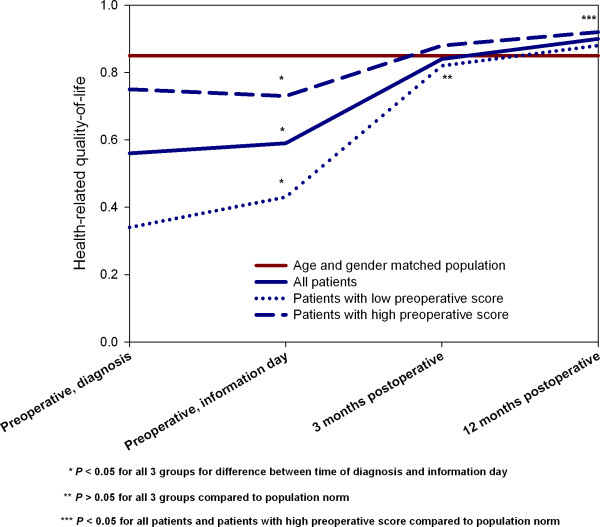
**Health-related quality-of-life (HRQOL) at the 4 time points for all patients and for patients with low and high preoperative score compared to the population norm**.

At the 3-months follow-up HRQOL had raised to 0.84 (SD, 0.14), which was not different from the population norm (*P *= 0.33). For the group with low (≤ 0.69) HRQOL at time of diagnosis, HRQOL was 0.82 (SD, 0.14), which was not different than the population norm (*P *= 0.06). For the group with high preoperative HRQOL, HRQOL was 0.86 (SD, 0.13), again not different from the population norm (*P *= 0.59) (Figure 1).

At the 12-months follow-up HRQOL exceeded the population norm with 0.90 (SD, 0.14) (*P *< 0.01). The group with low preoperative score had raised its average HRQOL to 0.88 (SD, 0.15), not different from the population norm (*P *= 0.12), whereas the group with a high preoperative score had an HRQOL of 0.92 (SD, 0.14), which was higher than the population norm (*P *< 0.01) Figure 1.

**Figure 2:**
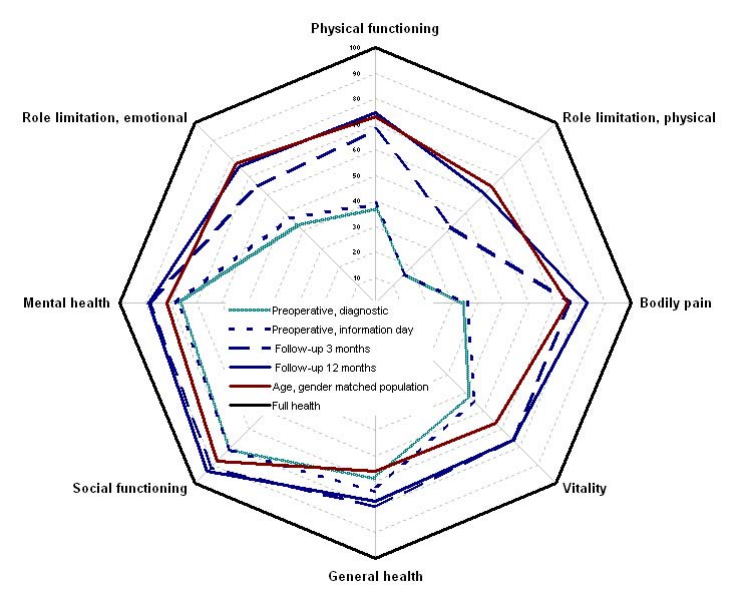
**The 8 dimensions of SF36 at the 4 time points compared to the population norm and full health**.

By using SF36, the age and gender matched mean population norm score for PF was 73.0. The age and gender specific population norm for all eight dimensions in SF36 is presented in Figure 2. The average PF for the patient sample preoperatively at time of diagnosis was 36.8 (SD, 20.6), preoperatively at the information day it was 39.0 (SD, 20.5) (*P *= 0.054 between time points).

At the 3-months follow-up the THA patients had reached the age and gender matched population norm for 5 of the 8 dimensions (bodily pain, vitality, general health, social functioning, and mental health) in SF36 (Figure 2). For PF we observed a mean value of 67.8 (SD, 19.1), which was lower than the population norm of 73.0 (*P *< 0.001). The observed score for role limitation due to emotional problems (RE) for the sample of 65.0 (SD, 41.3) was also lower (*P *< 0.001) than the population norm of 76.7. Likewise, the observed score for role limitation due to physical functioning (RP) of 42.3 (SD, 39.9) was lower than the population nom of 0.64 (*P *< 0.001). Compared to the population norm, the patient sample with low preoperative physical function (≤ 35) PF was 64.3 (SD, 19.2) (*P *< 0.001), and the sample with high preoperative score PF was 71.8 (*P *= 0.97).

At the 12-months follow-up the patients had reached a level at or above the population norm for all 8 dimensions in SF36. (RP with a mean score of 60.3 (SD, 42.6) (*P *= 0.26)) (Figure 2). For the patient sample with low preoperative physical function score (≤ 35) PF was 70.1 (SD, 21.6) at 12-months follow-up, which was not different from the population norm (*P *= 0.25).

### Disease specific outcome

The age and gender matched mean total HHS for the age and gender specific population was 94.0. The mean HHS at time of diagnosis was 45.7 (SD, 15.1), increased to 47.5 (SD, 15.0) (*P *= 0.02) at the information day.

**Figure 3:**
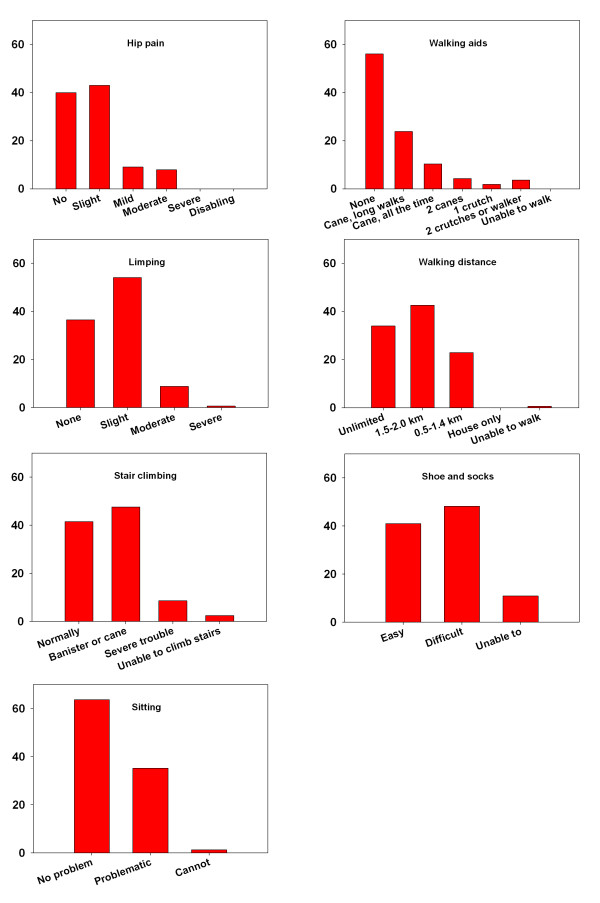
**Proportion of patients 3 months after fast-track THA answering each answer category of the 7 items included in self-report Harris Hip Score**.

At the 3-months follow-up visit mean HHS was 82.9 (SD, 13.1), lower than the population level (*P *< 0.001). A total of 20% of the patients had a score at or above the population level. The performance in each of the 7 items in HHS is presented in Figure 3. For the group with low (≤ 45) HHS at time of diagnosis mean HHS was 81.1 (SD, 14.0) at follow-up, lower than population level (*P *< 0.001), whereas 19% of the patients had a score at or above the population level. For the group with high HHS at time of diagnosis HHS was 84.3 (SD, 12.2), lower than population level (*P *< 0.001), whereas 23% of the patients had a score at or above the population level.

Compared to the population norm, at the 12 months follow-up mean HHS was 88.0 (SD, 15.1) (*P *< 0.001), however, 48% of the patients had a score at or above the population level. The group with low preoperative function had a mean HHS of 84.8 (SD, 18.1) (*P *< 0.001), and 41% of patients had a score at or above the population level, whereas the group with high score preoperatively had a mean HHS of 89.6 (SD, 12.5) (*P *= 0.01), of which 51% of patients had a score at or above the population level.

### Correlation between generic and disease specific outcomes

At 3 months postoperatively strong correlation was observed between HRQOL measured with EQ-5 D and disease specific score measures with HHS of 0.63. The regression analysis revealed an association with a coefficient of 0.007 (CI, 0.005 - 0.008) (*P *< 0.001), where 40% of the variance in HRQOL (R-squared) was explained by HHS. The dichotomized items most strongly associated with HRQOL in the final model was no or occasional use of cane 0.09 (CI, 0.02 - 0.16) (*P *< 0.01), walking distance above 1.5 km 0.05 (CI, 0.01 - 0.10) (*P *= 0.03), being able to easily put on socks and shoes 0.06 (0.02 - 0.09) (*P *< 0.01), and being able to sit in all chairs 0.11 (CI, 0.08 - 0.15) (*P *< 0.01). In total, 39% of the variance in PF (R-squared) was explained by these 4 items.

Likewise, 3 months postoperatively a strong correlation was observed between HRQOL measured with PF and disease specific score measures with HHS of 0.64. The regression analysis revealed an association with a coefficient of 0.92 (CI, 0.74 - 1.11) (*P *< 0.001), where 41% of the variance in HRQOL (R-squared) was explained by HHS. The dichotomized items most strongly associated with PF in the final model was the same as above no or occasional use of cane 9.7 (CI, 2.5 - 16.9) (*P *< 0.01), walking distance above 1.5 km 12.5 (CI, 5.9 - 19.1) (*P *< 0.01), being able to easily put on socks and shoes 5.9 (0.9 - 10.9) (*P *= 0.02), and being able to sit in all chairs 9.4 (CI, 3.9 - 14.8) (*P *< 0.01). In total, 36% of the variance in PF (R-squared) was explained by these 4 items.

At 12 months postoperatively significant correlation was observed between HRQOL measured with EQ-5 D and disease specific score measures with HHS of 0.80. The regression analysis revealed an association with a coefficient of 0.008 (CI, 0.007 - 0.009) (*P *< 0.001), where 64% of the variance in HRQOL (R-squared) was explained by HHS. A strong correlation 12 months postoperatively between HRQOL measured with PF and disease specific score measures with HHS of 0.70 was observed. The regression analysis revealed an association with a coefficient of 0.93 (CI, 0.76 - 1.09) (*P *< 0.001), where 49% of the variance in PF (R-squared) was explained by HHS.

## Discussion

To our knowledge this is the first study to present patient relevant long-term outcomes for patients following fast-track THA. The study reveals that patients following fast-track regain health within 3-12 months compared to an age and gender matched population group without any formal intensive postoperative rehabilitation when using generic HRQOL as an outcome. However, they do not regain health when using a disease specific outcome such as HHS.

For generic HRQOL outcome measured with EQ-5 D the patients as a whole reached a level that was comparable to the age and gender matched population norm at the 3-months follow-up, whereas they actually reached a level that was higher than the population norm at the 12-months follow-up. Even when sub-dividing the patients into groups with low and high preoperative HRQOL, the patients with low preoperative HRQOL had a non-significant lower HRQOL when compared to the population level at 3-months follow-up.

The Swedish Hip Arthroplasty Register (SHAR) is to our knowledge the only register, that monitor HRQOL by using EQ-5 D as a standard [28]. Our results for mean HRQOL of 0.90 after fast-track THA one year postoperatively, however, are higher than their reported average national value of 0.76, and also higher than the hospital with the highest average score, which was 0.86 [28]. This difference could be attributed to selection of patients into our fast-track intervention, but because the SHAR data resemble our data before implementing fast-track intervention [13,15,21,22] the difference in HRQOL may in our opinion mainly be caused by the fast-track intervention.

When using SF36 and looking at the PF, the results were somewhat different, because the patient group in general did not reach the population level at the 3-months follow-up. This was mostly explained by patients with low preoperative PF who did not reach the population norm, whereas patients with high preoperative PF were not different from the population norm. At the 12-months follow-up all patient groups had reached the population norm. The goal for a fast-track regimen should be to achieve the PF of the population norm as fast as possible and with as less pain and risk of complications as possible. In a fast-track context this goal should not be in 12 months but more likely in 3 months, and consequently we should in the future focus on the patient group with low preoperative function level which has the highest potential of improvement in order to shorten convalescence before 3-months follow-up within a fast-track context.

In contrast, by using the disease specific outcome HHS the patients never reached a level at the population level within one year postoperatively. This raises the principal question if we should introduce a further rehabilitation intervention for this patient group on the basis of generic outcome disease specific outcome or other outcomes. Thus, in the gender and age matched population level the average HRQOL is not 1 (Figure 1), but reduced from age itself and includes persons with different chronic diseases, such as diabetes, cardiac problems, respiratory problems, and other musculoskeletal problems that reduce the HRQOL. It is therefore questionable if it is reasonable to take this specific THA patient group with high HRQOL, but lacking full hip function, and raise their disease specific health state to a level that is at or above the age and gender specific norm at the cost of other patient groups with much lower HRQOL. We therefore propose that the ultimate goal after THA is to reach a HRQOL at the population level in general, with most focus at the pain and physical functioning level. One way to include disease specific outcome after fast-track THA is to set goals for the rehabilitation intervention and establish clinical indicators for pain and function so that a given proportion of patients at a given follow-up time have to reach a given level.

We have identified a strong general correlation between disease specific outcome and generic HRQOL outcomes that can be used to increase HRQOL by targeting those areas most strongly associated with HRQOL, which in this population were ability to walk without or only occasional use of cane, being able to walk 1.5 km or longer, being able to easily put on sock and shoes, and being able to sit comfortably in all chairs. These areas could easily be improved by postoperative rehabilitation.

In a fast-track context two strategies to improve patient outcome postoperatively immerges. One strategy is to focus on preoperative optimization of patients with low preoperative score measured with PF and to intervene with a preoperative physical optimization for this group. The current evidence of preoperative physical optimization is based on 4 randomized clinical trials [29-32]. All studies demonstrated a preoperative effect on pain and function, whereas the study by Gilmer et al. (2003) [30] was the only study to demonstrate a significant effect postoperatively by using a disease specific outcome. However, none of these studies were performed within the concept of fast-track surgery. The second strategy is to focus on early postoperative rehabilitation on those with specific problems, and intervene with a rehabilitation that can address these problems. However, the postoperative period is less feasible because postoperative mobilization restrictions hinder early and active rehabilitation. In our study, we used a conservative 3 months postoperative hip restriction period, which is a problem in fast-track because the restrictions interfere with recovery [33]. No consensus exists of postoperative hip restrictions, but the scarce existing evidence spreads from no restrictions to 6 weeks restrictions with no more than 90° of hip flexion, no adduction past neutral, and no internal rotation past neutral [33]. If the postoperative restriction could be omitted or strongly modified there is a great potential for early and aggressive rehabilitation intervention [34]. Another argument for being less restrictive postoperatively is the shift towards greater implant heads, which is thought to reduce the risk of dislocation.

In our follow-up period we had a total loss to follow-up of 5%, 15% at the 3-months follow-up and 23% at the 12 months follow-up. This proportion of loss to follow-up is normal in studies of this kind [23], but still a problem because loss to follow-up has been shown to be associated with both poorer or no difference in outcome [35,36]. We did, however, not observe any difference in the collected baseline variables between the patients who were followed up and the patients who were lost to follow-up and therefore consider our results to be unbiased and representative for the entire sample.

Another problem which has to be taken into account is the population norm scores we have used as controls. The data concerning EQ-5 D were obtained from three recent large Danish population studies including almost 26.000 persons providing very reliable data for comparison, but for SF36 the data were from 3950 persons and from 1994 [37,38] This is a possible flaw when comparing our results from 2007. However, until new and more precise population norm data are presented we believe that the SF-36 used population norm data is adequate and useful. For HHS no normative data exist from Denmark, and we used data from a study from California including only 184 persons aged 55 or more as controls, which also may give a flaw in our analysis.

Although we did create a rather homogeneous case mix for study purpose, we still believe that our observed results will apply for the most and average THA patients irrespective of age, gender and diagnosis.

## Conclusions

If HRQOL is considered the primary outcome after THA, the need for additional postoperative rehabilitation for all THA patients following a fast-track intervention is questionable. However, a pre- or early postoperative physical intervention seems relevant if the PF of the population norm should be reached at 3 months especially for those with low pre-operative functions, and this should be the goal for a fast-track regimen more than to reach the PF of the population norm after 12 months. If disease specific outcome is considered the primary outcome after fast-track THA, clear goals for the rehabilitation must be established before patient selection, intervention type and timing of intervention can be made.

## Competing interests

The authors declare that they have no competing interests.

## Authors' contributions

KL and TBH planned and performed the study. KL made the analysis and KL, TBH, KS and HK all contributed to interpretation of the analysis and preparation of the manuscript. All authors have read and approved the final manuscript.
